# Antioxidant activity of nanoencapsulated chia (*Salvia hispanica* L.) seed extract and its application to manufacture a functional cheese

**DOI:** 10.1002/fsn3.3169

**Published:** 2022-12-15

**Authors:** Farinaz Hosseini, Ali Motamedzadegan, Shahram Naghizadeh Raeisi, Somayeh Rahaiee

**Affiliations:** ^1^ Department of Food Science and Technology, Ayatollah Amoli Branch Islamic Azad University Amol Iran; ^2^ Department of Food Science and Technology Sari Agricultural Sciences and Natural Resource University Sari Iran; ^3^ Department of Microbial Biotechnology, Faculty of Biotechnology Amol University of Special Modern Technologies Amol Iran

**Keywords:** antioxidant activity, cheese, chia seed, nanoencapsulation, oxidative stability, *Salvia hispanica*

## Abstract

The study aimed to produce a functional ricotta cheese with chia seed extract (CSE) nanocapsules. First, the CSE was encapsulated using lecithin and basil seed gum, and its characteristics and antioxidant activity (AA) were evaluated. The free CSE (F‐CSE) and encapsulated CSE (E‐CSE) were then added to ricotta cheese formulation (1.5 and 3.0% w/w). The samples were kept for 15 days in a refrigerator and their physicochemical, sensory properties, AA, and oxidative stability were examined. The particle size, polydispersity index, zeta potential, and encapsulation efficiency of CSE nanocapsules were 59.23 nm, 0.328, −44.47 mV, and 80.06%, respectively. The CSE showed remarkable AA in vitro. The AA of F‐CSE was higher than E‐CSE. The moisture, dry matter, fat, and protein content of cheese samples were in the range of 52.64%–53.31%, 46.69%–47.36%, 19.02%–19.28%, and 16.88%–17.02%, respectively. The color of F‐CSE cheeses was slightly yellower than control; however, they did not have clear color differences. During storage, the acidity, hardness, chewiness, and peroxide value of cheeses increased, while the pH, total phenol content, and AA decreased (*p* < .05). The addition of CSE reduced the rate of pH and acidity changes during storage and significantly increase the AA and oxidative stability. Initially, F‐CSE cheeses had higher functional activity, but on other storage days, due to the protective effect of coating materials, the functional activity of E‐CSE samples was higher. The CSE, especially E‐CSE, did not have an adverse effect on the sensory properties of cheese. Based on the results of this study, it can be concluded that it is possible to manufacture a functional cheese using E‐CSE.

## Graphical abstract

We encapsulated the chia seed extract (CSE) nanoliposomes with basil seed gum (BSG) successfully. The free CSE (F‐CSE) and encapsulated CSE (E‐CSE) were added to ricotta cheese formulation (1.5% and 3.0% w/w) to produce a functional cheese. The E‐CSE cheeses had higher functional activity, during 15 days storage, comparing the E‐CSE samples.

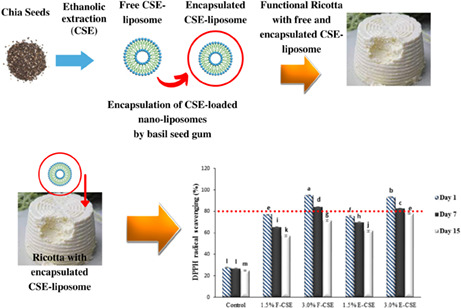

## 1 INTRODUCTION

Ricotta cheese is one of the popular types of unripe and fresh cheeses around the world which contains high amounts of moisture. It is obtained from whey, whole milk powder, skim milk, or a mixture of whey and whole milk. The ricotta cheese production process includes acidification with citric acid or lactic acid, and a heating process at a temperature of 85°C–90°C for 20–30 min to coagulate the proteins of whey (Nzekoue et al., 2021; Rubel et al., 2019). This type of cheese has a slightly sour taste, yellowish‐white color, thick, grainy and soft texture, and contains various nutrients. Ricotta cheese is high in protein and low in fat and calorie, so it is recommended for obese or overweight people (Asensio et al., 2014). The process of enrichment with various functional and bioactive agents (like dietary fiber, antioxidants, and vitamins) can improve the nutritional quality and health benefits of food products (Siyar et al., 2022). Today, the demand for food products with bioactive compounds is increasing (Alemán et al., 2021; Selahvarzi, Ramezan, et al., 2021; Selahvarzi, Sanjabi, et al., 2021). Dairy products, especially cheeses and yogurt, are considered as a suitable and good carriers for functional compounds (Picciotti et al., 2021).

Bioactive substances such as polyphenols are found naturally and abundantly in various plant sources, including vegetables, cereals, fruits, roots, pulses, and so on (Li et al., 2018). The main bioactive compounds of these natural sources are flavonoid and phenolics, which are known for their health benefits (Gulcin, 2012). A lot of variety of phenolic compounds containing one or more aromatic rings are in plants food and responsible for flavor, color, and texture. The simpler phenolic materials include monophenols found in fruits and seeds, thehydroxycinnamic acid groups, flavonoids, and flavonols (Gulcin, 2020). Some of these compounds are known to be powerful antioxidants that can protect the human body against diseases caused by oxidative stress and damage (Croft et al., 2018). Chia (*Salvia hispanica* L.) is an herbaceous plant that belongs to the *Lamiaceae* mint family which is native to Mexico and Guatemala (Kuznetcova et al., 2020). Chia seeds are rich in protein, omega‐3 fatty acids, dietary fiber, minerals, vitamins, and polyphenol antioxidants, and also have various health benefits such as improving digestion, cleansing the colon, improving brain and heart health, improving the immune system, regulating blood sugar, and so on (Hawaldar & Ballal, 2021). Chia seeds have been considered as functional components (Nduko et al., 2018). The presence of high levels of various polyphenols such as chlorogenic acid, caffeic acid, rosmarinic acid, querencetin, gallic, myricetin, cinnamic acid, and kaemferol has been reported in chia seeds (Knez Hrnčič et al., 2020).

However, due to the presence of numerous unsaturated bonds in the structure of plant bioactive substances, most of these bioactive compounds were unstable and degradable rapidly when exposed to heat, oxygen, and light. Encapsulation is an effective technique to increase the stability of sensitive bioactive substances as well as their controlled release over time (Bai et al., 2021). Encapsulation is a process in which the active and sensitive substances are covered and trapped by carriers of different materials causing their controlled release. They can improve the stability of compounds against environmental stressors (such as moisture, radiation, oxygen, light, and adverse pH conditions) as well as against digestive conditions in the body (Timilsena et al., 2020; Wardhani et al., 2021). This technology has tremendous applications in both the pharmaceutical and food industries (Tavakoli et al., 2021). It is classified into two categories based on the scale of particles produced: micro‐capsulation (particles with sizes from 1 to 100 micrometers) and nano‐encapsulation (particles with sizes from 10 to 1000 nanometers). Both methods are used to improve the performance and efficiency of active compounds (Shishir et al., 2018). Research is increasing to find new and alternative sources for the proper delivery of these active compounds such as antioxidants, antimicrobials, probiotics, etc. (Abedinia et al., 2020, 2021). Various materials including carbohydrates, gums, proteins, and lipids are used as wall materials for the production of the capsules, and the type of materials used affects the efficiency of encapsulation and the release of active compounds (Meng et al., 2021).

The delivery systems based on lipids such as liposomes and nano‐liposomes are the widely used technologies to increase the bioavailability, bio‐accessibility, stability, solubility, and stability of phenolic substances. These systems are vesicles that are composed of single or multiple layers of phospholipids that have both hydrophilic and hydrophobic parts and therefore are used to encapsulate bioactive compounds with different lipophilic, hydrophilic, and amphiphilic nature (Azarashkan et al., 2022). Different methods are used to prepare liposomes, including solvent evaporation, thin‐film dehydration, electroformation, proliposome, dialysis, sonication, membrane extrusion, extrusion, micro‐fluidization, high‐pressure homogenization, and freeze‐thawing (Isailović et al., 2013). Basil (*Ocimum basilicum*) is a common edible plant that has a unique flavor and aroma and contains noticeable amounts of gum or mucilage. It is well known as flavoring principles and it has known to contain antioxidants phenolic compounds such as rosmarinic acid (Gulcin et al., 2007). Basil seed gum (BSG) contains carbohydrates (79.6%), lipid (9.7%), moisture (8.1%), ash (3.3%), protein (1.6%), and starch (1.53%) (Hosseini‐Parvar et al., 2010). The BSG is inexpensive, available, and non‐toxic and exhibits excellent physicochemical properties such as high viscosity, high water‐absorbing ability, shear‐thinning behavior, pseudoplastic behavior, heat resistance (Maqsood et al., 2020), and stabilizing and emulsifying properties (Biglarian et al., 2021). Previous studies have shown that BSG can be used as a suitable wall material for the encapsulation of bioactive compounds (Shaygannia et al., 2021).

To the best of our knowledge, chia seed extract (CSE) has not been studied to fortify cheese types like the popular dairy product. Therefore, this study aimed to enrich the ricotta cheese formulation using free and nano‐encapsulated CSE and to investigate the physicochemical, textural, antioxidant, and sensory properties of the produced cheese.

## 2 MATERIALS AND METHODS

### 2.1 Materials

Chia seeds and BSG powder were obtained from a local market (Sari, Iran) and the Science and Technology Park laboratory of Sari (Iran), respectively. Lecithin (with 99% purity) was purchased from America Across Company. Whole milk powder was obtained from Golshad Ltd. (Iran). The analytical grade chemicals used in the research were obtained from Sigma‐Aldrich Company (USA).

### 2.2 Preparation of CSE

To prepare the CSE, first, the dried chia seeds were pulverized and then 50 g of the powder was added to 50% ethanol solvent. The powder was thoroughly mixed with the solvent and stirred on a magnetic stirrer at room temperature for 24 h. The obtained extract was then filtered (Whatman No. 41) and concentrated by a rotary evaporator. Finally, it was freeze‐dried (Operon FDB‐550) (Kwon et al., 2019).

### 2.3 Preparation of CSE‐loaded nano‐capsules

To prepare CSE nano‐capsules, first, its nano‐liposomes were prepared and then these nano‐liposomes were coated with different concentrations of BSG. To prepare the CSE‐loaded nano‐liposomes by thin‐film hydration method (Pinilla & Brandelli, 2016), first, a certain amount of CSE (to reach the final level of 0.5% w/w dry matter) were dissolved in a lecithin/chloroform mixture (in a 1:1 ratio), and then the solvent was removed using a rotary evaporator (Strike 300) for 10 min at 45°C. Then, it was dried by a vacuum oven (Memmert) at 40°C. The micrometer scale liposomes had a multilayered structure. To convert them to nanometer scale, the liposomal films were dissolved in 20 ml of 10 mM phosphate buffer solution (pH 7) and then sonicated at 400 Watts by a probe sonicator (2 cycles of 2 min with 10 s rest between cycles) (Hiescher, UP200H). The mono‐layered liposomes at the nanometer scale were obtained. We considered different levels of extract (0.5, 1, 1.5%) and gum (0.5, 1, 1.5%) and choose the best treatment and used in this study (data not shown).

To coat the CSE‐loaded nano‐liposomes by BSG, first, the solution of 1% w/w BSG was obtained by dissolving it in 20 ml of deionized water and then stirring at 1500 rpm and keeping at the refrigerator overnight. The BSG solution was added dropwise to the CSE liposome suspension (an equal volume) and stirred for 2 h (at 800 rpm). Finally, the CSE capsules were dried by a freezing dryer (Operon FDB‐550) for 19 h at −70°C and then pulverized.

### 2.4 The characteristics of CSE‐loaded nano‐capsules

The mean particle size, polydispersity index (PDI), and zeta potential of the samples were determined by dynamic light scattering (DLS) using a Zetasizer (nano‐Zs). For the initial preparation of capsules samples, the samples were diluted with distilled water up to 10 times their volume. The obtained data were collected and analyzed using Zetasizer data BIC Particle Sizing Software (Shahavi et al., 2015). To determine the zeta potential, the nano‐capsule sample was diluted with distilled water (at 50 times its volume), and the amount of zeta potential was determined at the power of 149 W, pH 7.4, and temperature of 25°C.

The total phenol content (TPC) of the capsule samples was evaluated to determine the encapsulation efficiency (EE). For EE, 200 mg of capsule sample was added to 2 ml of a mixture of methanol–acetic acid–water (ratio of 50:8:42 v/v/v), stirred for 1 min, and ultrasound was performed in two cycles for 20 min at a frequency of 20 kHz. It was then centrifuged at 3500 *g* for 10 min and the TPC in the upper solution was measured using the Folin–Ciocalteu method (Gulcin et al., 2011). The initial TPC was also determined, and through the following equation, the EE% of samples were obtained (Robert et al., 2010):

$$\mathrm{EE}\%=\left(\text{the}\;\mathrm{TPC}\;\text{contained in the capsules}/\text{the initial}\;\mathrm{TPC}\right)\times 100$$

### 2.5 Antioxidant activity of CSE loaded‐nanocapsules

The antioxidant activity (AA) of free (F‐CSE) and encapsulated (E‐CSE) CSE were measured by two methods of 2,2‐diphenyl‐1‐picrylhydrazyl (DPPH) radical scavenging, and ferric reducing antioxidant power (FRAP). The DPPH radical scavenging was measured according to the method expressed by Ruslan et al. (2018) with some modifications. Briefly, 2 ml of methanolic extract sample was mixed with 0.1 mM DPPH solution. The resulting mixture was stirred well and kept in the dark at room temperature (23 ± 2°C) for 30 min. The absorbance of sample solution was recorded at 515 nm against blank. The DPPH radical scavenging activity (%) of extracts was calculated through the following equation. Ac and As are the absorbance of blank and sample at 517 nm, respectively:

$$\text{DPPH radical scavenging}\;\left(\%\right)=\frac{\mathrm{Ac}-\mathrm{As}}{\mathrm{Ac}}\times 100$$

to evaluate the AA of extracts by FRAP, briefly, 30 μl of the sample was mixed with 90 μl of distilled water and 900 μl of a working solution containing 25 ml of 300 mM acetate buffer, 2.5 ml of 20 mM FeCl3·6H2O solution, and 2.5 ml of TPTZ solution in HCl (40 mM), and kept for 30 min at 37°C. After that, the absorbance of the solution was recorded at 595 nm. FRAP values of samples were obtained through the standard curve (absorbance of different concentration of Trolox including 300 mM–1500 mM) (Beltrán‐Orozco et al., 2020).

### 2.6 Manufacture of ricotta cheese

To manufacture ricotta cheese samples, a mixture of whole milk powder and water (40:60) was first prepared and its pH was reached to 7 (with 1 N sodium hydroxide), and then it was heated for 30 min at a temperature of 90°C. Lactic acid was added to the mixture at a level of 25 ml/L and a curd was formed which was collected in a mold with dimensions of 43 mm × 20 mm. After separating the curd from the whey, the prepared cheese was cooled to room temperature (Pontonio et al., 2021). The F‐CSE and E‐CSE were added in levels of 1.5% and 3.0% w/w in the cheese samples and mixed for 5 min. The control cheese was without CSE. The prepared cheeses were kept in the refrigerator (4°C) for 15 days.

### 2.7 Compositional and physicochemical analyses of cheeses

The chemical composition of cheeses, including moisture, fat, protein, dry matter, and ash contents, was determined by AOAC standard method (AOAC, 2000). To determine the moisture content, about 5 g was dried at 105°C until it reached a constant weight. The fat content of cheese samples was measured using the method of Gerber‐Van Gulik. To determine the protein content, the total nitrogen content was first measured using the method of Kjeldahl and their values were converted into protein amount by the conversion factor of nitrogen to protein for dairy products (6.38). The ash content was measured using an electric furnace. The amount of total dry matter was obtained by subtracting the moisture content from 100. The pH and titratable acidity (% lactic acid) amounts of cheeses were determined using a digital pH meter (Pontonio et al., 2021) and titration with 0.1 sodium hydroxide (Abebe & Emire, 2020), respectively.

### 2.8 Color analysis of cheeses

The color parameters, including L* (lightness), a* (red or green color), and b* (yellow or blue color) were investigated in the Colorflex Hunter Lab. The sample was placed into the colorimeter device and photographed, and the L*, a*, and b* color parameters values were recorded (Abebe & Emire, 2020).

### 2.9 Texture analysis of cheese

The textural parameters, including hardness, cohesiveness, springiness, and chewiness, were measured at room temperature (23 ± 2°C) using a texture analyzer equipped with a flat glass probe. The weight of the sample, speed of test, and sample deformation were 60 g, 1 mm/s, and 30%, respectively. Two compression cycles were used (Pontonio et al., 2021).

### 2.10 TPC of cheeses

To prepare the extract of each cheese sample, cheese (5 g) was mixed with methanol solvent (5 ml) for 5 min. The extract was placed for 2 h at 4°C and then centrifuged at 8602 × g, 4°C for 30 min. Finally, the cheese extracts were filtered by Whatman No.42 (Akan et al., 2021).

The TPC was measured using the Folin–Ciocalteu method and according to the method expressed by Pimentel‐González et al. (2015). For this purpose, a mixture of 1 ml extract and 5 ml of Folin–Ciocalteau reagent was prepared. After stirring for 6 min, 20% sodium carbonate (4 ml) was added to the mixture and kept at 23°C for 120 min. The absorbance of the mixture was then measured at 760 nm. The cheese TPC was expressed as mg Gallic acid equivalents/g sample (mg GAE/g sample).

### 2.11 AA of cheeses

The AA of ricotta cheese samples was measured by DPPH radical scavenging and FRAP methods. The methods of measuring these two antioxidant tests were following the expressed methods for measuring the AA of free and encapsulated CSE.

### 2.12 Measurement of peroxide value of cheeses

First, the fat of samples was extracted by mixing 10 g of cheese with 40 ml methanol and 20 ml chloroform for 2 min. Then, 20 ml methanol and 20 ml chloroform were added to the mixture. Finally, it was filtrated (Whatman No. 1) and then the solvent was evaporated by the rotary evaporator.

To evaluate the peroxide value (PV), the extracted fat (0.1 g) was dissolved in a mixture of acetic acid/chloroform (25 ml; 3:2 v/v ratio). After that, 1 ml potassium iodide was added to the mixture, and the mixture was kept for 10 min in a dark place. Twenty milliliters of distilled water was then poured and was titrated with sodium thiosulfate in the presence of starch solution (1.5%). The PV was reported as meq/kg sample (Saravani et al., 2019).

### 2.13 Sensory evaluation of cheeses

The sensory characteristics were studied using a 5‐point Hedonic test (1: very bad and 5: very good) by 10 panelists (five male and five female). Cheese samples at ambient temperature and in equal weight were poured into coded plastic containers and given to the panelists and were scored in terms of flavor, color, odor, appearance, and overall acceptance.

### 2.14 Statistical analysis

Statistical analysis was done in triplicate for all the samples and experiments, and the obtained data are demonstrated as mean ± SD. The one‐way ANOVA analysis, Duncan multi‐range post hoc test, and IBM SPSS Statistics 22.0 were used to analyze the data at significance level of *p* < .05.

## 3 RESULTS AND DISCUSSION

### 3.1 Chia seed extract‐loaded nanocapsules characterization

Particle size and PDI are important parameters in evaluating the colloidal system characteristics, especially the stability and EE (Fathi et al., 2012). The PDI of nanoparticles is in the range of zero to one, and the PDI values > 0.5 indicate broader size distribution (Tamjidi et al., 2013). The mean particle size and PDI values of the CSE‐loaded nanocapsule are presented in Table 1. The particle size and PDI of nanocapsules were 59.23 nm and 0.328, respectively. The EE is also another important parameter to determine encapsulation effectiveness. In this test, the number of bioactive compounds trapped in the core of the capsules is measured. Generally, the encapsulation process will have the highest EE, if almost all polyphenols or bioactive compounds are completely entrapped inside the wall materials and improve the bioactive substance stability. The CSE nanocapsules indicated a high EE (80.06%). In previous studies, different values of particle size, PDI, and EE have been reported for active compounds coated with BSG. For example, Rashidaie Abandansarie et al. (2019) reported that the particle size, PDI, and EE of rosemary extract capsules prepared with BSG were 154.9 nm, 0.140, and 58.71%, respectively. Komijani et al. (2020) reported the EE of lycopene encapsulated with BSG and polyvinyl alcohol in the range of 80.04%–91.67%, which was comparable to the efficiency obtained in this study.

**TABLE 1:** Characteristics of chia seed extract (CSE)‐loaded nano‐capsules

Sample	Particle size (nm)	PDI index	Encapsulation efficiency (%)	Zeta potential (mV)
CSE‐loaded nanocapsule	59.23 ± 1.75	0.328 ± 0.017	80.06 ± 0.53	−44.47 ± 0.28

*Note*: Values represent mean (*n* = 3) ± SD. Different letters in each column represent significant difference at 5% level of probability among samples.

The zeta potential indicates the emulsion stability and the surface electric charge of the particles (Premi & Sharma, 2017). High values of zeta potential indicate higher stability of systems against sedimentation, and the desirable zeta potential value is above 30 mV (Mozafari et al., 2008). The CSE‐loaded nanocapsules had a zeta potential of −44.47 mV (Table 1), so they indicated good stability. Research has shown that because gums contain carboxylic acid groups, they have negative zeta potential (de Campo et al., 2017). However, Fırtın et al. (2020) reported lower zeta potential values for chia seed oil capsules prepared with maltodextrin and Arabic gum (−32.2 to −40.6 mV). The saffron bioactive compounds encapsulated with BSG and whey protein concentrate had −74 mV zeta potential (Gahruie et al., 2020).

### 3.2 Antioxidant activity of F‐CSE and E‐CSE

The AA was measured by two methods of DPPH radical scavenging activity and FRAP. The DPPH test is a quick and simple method to determine the AA of food components and indicates the ability of an active compound to donate atoms of hydrogen to unstable free radicals. The reduction sufficiency of DPPH radicals specified by the decrease in its absorbance at 517 nm induced by antioxidants. It causes a change in color from purple to yellow. Therefore, DPPH is used for evaluate the activity of antioxidants (Elmastas et al., 2006). In the FRAP method, the reductive capacity of Fe (III) (ferric iron) to Fe (II) (ferrous iron) is measured (Dravie et al., 2020; Esmaeilzadeh Kenari & Razavi, 2022). The AA of free and encapsulated CSE is given in Table 2. As the results demonstrate that the DPPH and FRAP values for F‐CSE were 50.48% and 69.55 μmol Trolox equivalents (TE)/g of sample, respectively, and these values for E‐CSE < F‐CSE (46.02% and 67.92 μmol TE/g of the sample, respectively). Relatively higher AA of free extracts than encapsulated extracts has also been confirmed by some other researchers (Esmaeilzadeh Kenari & Razavi, 2022; Savaghebi et al., 2020). Beltrán‐Orozco et al. (2020) also studied the AA of chia seeds by DPPH, ABTS, and FRAP methods and reported its amounts of 41.1, 77.7, and 72.3 μmol TE/g of the sample, respectively. The researchers attributed the remarkable AA of chia seeds to the presence of significant values of bioactive compounds such as polyphenols, vitamin C, and flavonoids. Significant AA of chia seeds has been observed in other previous studies (Amato et al., 2015; Scapin et al., 2016).

**TABLE 2:** Antioxidant activity of free and encapsulated CSE

Samples	DPPH (%)	FRAP (μmol TE/g sample)
F‐CSE	50.48 ± 0.69^a^	69.55 ± 0.85^a^
E‐CSE	46.02 ± 0.94^b^	67.92 ± 0.72^b^

*Note*: Values represent mean (*n* = 3) ± SD. Different letters in each column represent significant difference (5% level).

Abbreviations: CSE, chia seed extract; E‐CSE, encapsulated CSE; F‐CSE, free CSE; FRAP, ferric reducing antioxidant power.

### 3.3 Chemical composition of cheeses

The results are given in Table 3. The results demonstrated that the addition of F‐CSE did not have a significant effect on the moisture and dry matter content of cheeses, while the E‐CSE increased the moisture content and decreased the dry matter content significantly (*p* < .05) so that the moisture content and dry matter content of the control cheeses and F‐CSE samples were in the range of 52.64%–52.80% and 47.20%–47.36%, respectively, and in the E‐CSE cheeses reached to 53.18%–53.31% and 46.69%–46.82%, respectively. The presence of BSG in the coating of CSE nanocapsules is one of the major reasons for increasing the moisture content and reducing the dry matter due to the addition of CSE‐loaded nanocapsules, because gums can absorb and retain high amounts of moisture (Mudgil, 2021). On the other hand, the liposomal membrane is able to bind water on its surfaces (Laloy et al., 1998). However, the addition of free and encapsulated CSE did not show a significant effect on the fat and protein content. The fat and protein content of samples were in the range of 19.02%–19.28% and 16.88%–17.02%, respectively.

**TABLE 3:** Chemical composition of ricotta cheese samples

Samples	Moisture content (%)	Protein content (%)	Fat content (%)	Dry matter (%)
Control	52.64 ± 0.27^b^	16.88 ± 0.21^a^	19.02 ± 0.13^a^	47.36 ± 0.27^a^
1.5% F‐CSE	52.78 ± 0.23^b^	16.97 ± 0.15^a^	19.17 ± 0.10^a^	47.22 ± 0.23^a^
3.0% F‐CSE	52.80 ± 0.17^b^	16.99 ± 0.24^a^	19.24 ± 0.07^a^	47.20 ± 0.17^a^
1.5% E‐CSE	53.18 ± 0.15^a^	17.01 ± 0.13^a^	19.19 ± 0.11^a^	46.82 ± 0.15^b^
3.0% E‐CSE	53.31 ± 0.21^a^	17.02 ± 0.18^a^	19.28 ± 0.15^a^	46.69 ± 0.21^b^

*Note*: Values represent mean (*n* = 3) ± SD. Different letters in each column represent significant difference (5% level).

Abbreviations: CSE, chia seed extract; E‐CSE, encapsulated CSE; F‐CSE, free CSE.

According to Hamdy and Hafaz (2018), there was no significant difference between the moisture, protein, and fat content of control ricotta cheese and samples fortified with rosemary, basil, and thyme herbs. The amounts of fat (15.00%–15.18%) and the protein (11.48%–11.57%) reported in their study were lower than the amount obtained in this study, but they obtained a higher moisture content (69.11%–69.32%), which is probably related to the type of raw material used to cheese manufacture. In their research, whey was used, while in this study, whole milk powder was used. Siyar et al. (2022) also indicated that adding different levels of nanoliposomal saffron extract did not have a significant effect on the protein, fat, and ash content of ricotta cheese; however, at high levels of nanoliposomes, there was an increase in moisture content and a decrease in dry matter amount, which was in accordance the results of this study. Jeong et al. (2017) also found that the incorporation of tomato extract microcapsules had no significant effect on the chemical composition of the Queso Blanco cheese.

### 3.4 pH and acidity of cheeses

**FIGURE 1:**
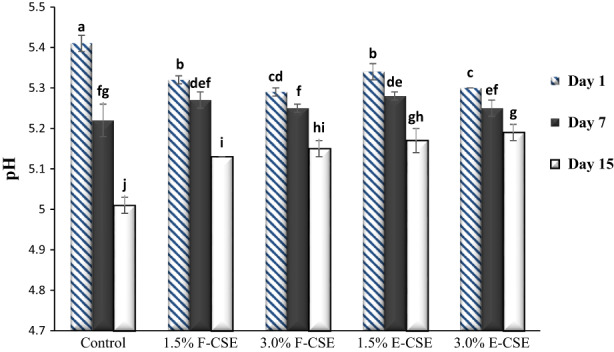
The pH of the ricotta cheese samples during 15‐day refrigerated storage period. Bars represent mean (*n* = 3) ± SD. Different letters on the bars indicate significant difference at 5% level of probability among samples. CSE, chia seed extract; E‐CSE, encapsulated CSE; F‐CSE, free CSE.

**FIGURE 2:**
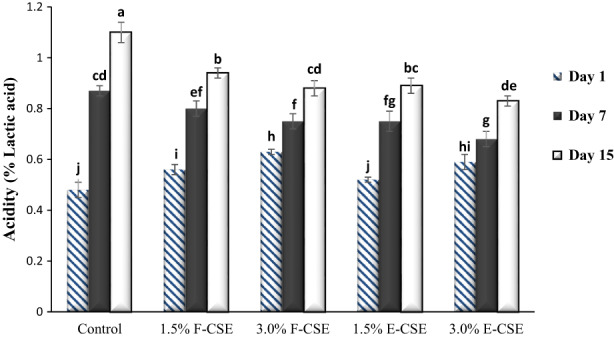
The titratable acidity of the ricotta cheese samples during 15‐day refrigerated storage period. Bars represent mean (*n* = 3) ± SD. Different letters on the bars indicate significant difference at 5% level of probability among samples. CSE, chia seed extract; E‐CSE, encapsulated CSE; F‐CSE, free CSE.

Changes in pH and acidity values of F‐CSE and E‐CSE cheese samples during the 15‐day storage period in the refrigerator are shown in Figures 1 and 2, respectively. On the first day of experiments, the incorporation F‐CSE and E‐CSE significantly reduced the pH and increased the acidity of samples compared to control (*p* < .05). It is probably due to the presence of acidic compounds in this extract. During the storage, the pH values decreased and the acidity increased (*p* < .05) so that the pH and acidity values of cheeses on the first day of experiments were in the range of 5.30%–5.41% and 0.48–0.63%, respectively, and on the last day reached to 5.01%–5.19% and 0.83%–1.10%, respectively. This is associated with the degradation of sugars and production of the acidic compounds (especially lactic acid) (Hala et al., 2010). The addition of CSE by reducing the activity of microorganisms in cheese samples could reduce the rate of pH and acidity changes during the storage, which is in line with the results of other researchers (El‐Galeel et al., 2018). Decreased pH of cheeses due to the incorporation of encapsulated tomato extract and liposomal saffron extracts has also been observed by Jeong et al. (2017), and Siyar et al. (2022), respectively. A decrease in the pH of ricotta cheese and an increase in its acidity during storage period were also reported by Souza et al. (2016). In research conducted by Hamdy and Hafaz (2018), the addition of different herbs as well as increasing the storage period decreased the pH values of ricotta cheese samples.

### 3.5 Color parameters of cheeses

Color is one of the most effective parameters on the acceptance of produced products by the consumers; hence, it should be noted that the functional additives used in the formulation of food products do not have an adverse effect on their color (Prudêncio et al., 2014). Table 4 shows the color parameters of the functional samples. The addition of CSE reduced the lightness and increased the yellowness of cheeses compared to the control. Color changes in F‐CSE samples were significant compared to the control (*p* < .05); however, there was no statistically significant difference between the color parameters of the control sample and E‐CSE cheeses. The L* and b* values of different samples were in the range of 87.58–90.48 and 24.62–26.71, respectively. Adding the CSE had no significant effect on the a* index (−2.20 to −2.25). The CSE used in this study had a creamy color, so the slight increase in the yellowness of the samples due to the incorporation of CSE was not unexpected. These results are in agreement with those observed by El‐Messery et al. (2019), who found that processed cheese containing mandarin peel extract nanoliposomes had a lower lightness and higher yellowness than control cheese. The study conducted by Kwon et al. (2019) showed that the addition ethanol extract of CSE did not have a significant effect on the lightness of yogurt and only increased the yellowness. Siyar et al. (2022) reported a decrease in lightness and increase in greenness and yellowness in ricotta cheese samples containing nanoliposomal saffron extract compared to the control sample and attributed the cause of these color changes to the presence of natural pigments such as chlorophyll and carotenoids in the saffron.

**TABLE 4:** Color parameters of ricotta cheese samples

Samples	L*	a*	b*
Control	90.48 ± 0.86^a^	−2.25 ± 0.09^a^	24.62 ± 0.49^c^
1.5% F‐CSE	88.73 ± 0.74^c^	−2.25 ± 0.06^a^	25.79 ± 0.37^b^
3.0% F‐CSE	87.58 ± 0.68^c^	−2.34 ± 0.07^a^	26.71 ± 0.52^a^
1.5% E‐CSE	90.16 ± 0.89^ab^	−2.20 ± 0.04^a^	24.66 ± 0.43^c^
3.0% E‐CSE	89.81 ± 0.77^ab^	−2.20 ± 0.07^a^	25.10 ± 0.40^bc^

*Note*: Values represent mean (*n* = 3) ± SD. Different letters in each column represent significant difference (5% level).

Abbreviations: CSE, chia seed extract; E‐CSE, encapsulated CSE; F‐CSE, free CSE.

### 3.6 Texture parameters of cheeses

The texture is another important parameter that has a noticeable effect on the acceptance of food products by consumers. In general, it depends on the sample's microstructure, and the chemical composition such as fat, moisture, total solids, and salt content (Souza et al., 2016). Textural parameters of samples including hardness, springiness, cohesiveness, and chewiness are given in Table 5. The addition of CSE did not show a significant change in cohesiveness and springiness of ricotta cheeses; however, it reduced the hardness and chewiness values compared to the control sample (*p* < .05). The hardness and chewiness values of the control cheese were 97.08 and 93.44 g/mm, respectively, and in cheeses containing CSE decreased to 91.07–94.35 g and 90.34–92.03 g/mm, respectively. During the storage period, no significant change was observed in the cohesiveness and springiness of cheese texture, while their hardness and chewiness gradually increased (*p* < .05), which is probably related to the decrease in moisture content of samples and pH changes over time. Previous researchers have also stated that polyphenols affect the textural parameters of the curd net by affecting the pH value, moisture, and soluble calcium content (Perez‐Gregorio & Simal‐Gandara, 2017). So that increasing the moisture content and decreasing the pH values of cheeses can soften the texture of the samples. In agreement with the results of this study, Borhanpour et al. (2022) found that the hardness value of cheese samples containing microencapsulated chavil extract was lower than that of chavil extract. Souza et al. (2016) also reported that there was no significant change in the texture parameters of ricotta cheese including hardness, cohesiveness, elasticity, gumminess, and chewiness during the 22‐day storage period. However, in the study on the effect of nanoliposomal saffron extract on the textural parameters of ricotta cheese, Siyar et al. (2022) observed that the incorporation of nanoliposomes increased the hardness and chewiness of cheeses, but did not show a significant effect on cohesiveness, adhesiveness, and gumminess of samples.

**TABLE 5:** Textural parameters of the ricotta cheese samples

Samples	Storage period (day)	Hardness (g)	Cohesiveness	Springiness (mm)	Chewiness (g/mm)
Control	1	97.08 ± 0.92^h^	0.26 ± 0.05^a^	6.990 ± 0.003^a^	93.44 ± 0.66^bc^
	7	129.16 ± 0.74^c^	0.27 ± 0.03^a^	6.992 ± 0.003^a^	94.62 ± 0.83^ab^
	15	149.65 ± 1.57^a^	0.29 ± 0.05^a^	6.990 ± 0.004^a^	95.82 ± 0.64^a^
1.5% F‐CSE	1	94.35 ± 1.10^i^	0.27 ± 0.04^a^	6.991 ± 0.002^a^	92.03 ± 0.60^def^
	7	116.53 ± 1.61^e^	0.28 ± 0.03^a^	6.995 ± 0.004^a^	93.60 ± 0.94^bcd^
	15	132.63 ± 0.92^b^	0.31 ± 0.04^a^	6.991 ± 0.003^a^	94.52 ± 0.46^b^
3.0% F‐CSE	1	92.05 ± 1.31^ij^	0.28 ± 0.04^a^	6.994 ± 0.005^a^	90.34 ± 0.80^g^
	7	115.00 ± 1.19^ef^	0.30 ± 0.03^a^	6.994 ± 0.003^a^	91.26 ± 0.57^efg^
	15	132.09 ± 0.81^b^	0.29 ± 0.05^a^	6.990 ± 0.004^a^	92.44 ± 0.68^cde^
1.5% E‐CSE	1	93.72 ± 0.99^i^	0.31 ± 0.02^a^	6.994 ± 0.005^a^	91.15 ± 0.89^efg^
	7	113.58 ± 1.03^f^	0.29 ± 0.04^a^	6.993 ± 0.004^a^	92.31 ± 0.82^cde^
	15	129.27 ± 1.49^c^	0.30 ± 0.04^a^	6.991 ± 0.006^a^	93.65 ± 0.62^bc^
3.0% E‐CSE	1	91.07 ± 0.93^j^	0.31 ± 0.06^a^	6.991 ± 0.005^a^	90.85 ± 0.71^fg^
	7	110.89 ± 0.86^g^	0.29 ± 0.05^a^	6.992 ± 0.003^a^	91.44 ± 0.61^efg^
	15	126.16 ± 1.02^d^	0.30 ± 0.03^a^	6.996 ± 0.004^a^	92.85 ± 0.67^cd^

*Note*: Values represent mean (*n* = 3) ± SD. Different letters in each column represent significant difference at 5% level of probability among samples.

Abbreviations: CSE, chia seed extract; E‐CSE, encapsulated CSE; F‐CSE, free CSE.

### 3.7 Total phenol content of cheeses

**FIGURE 3:**
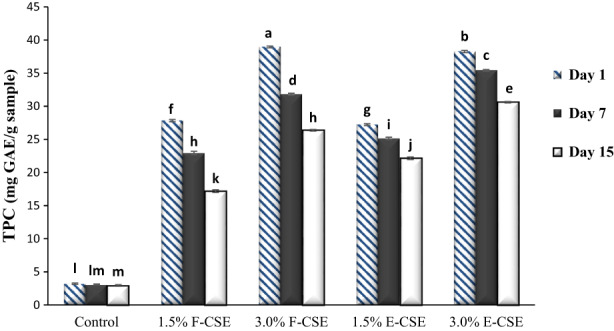
The total phenol content (TPC) of the ricotta cheese samples during 15‐day refrigerated storage period. Bars represent mean (*n* = 3) ± SD. Different letters on the bars indicate significant difference at 5% level of probability among samples. CSE, chia seed extract; E‐CSE, encapsulated CSE; F‐CSE, free CSE.

Polyphenols are a large group of phytochemicals that are found naturally in plant sources and contain one or more phenol units in their structure (Saphier et al., 2017). They exist in the aerial parts of plants such as flower, leaves, fruits, and seeds. Increasing attention to phenolic compounds because of their biological attraction, it has diverted properties such as AA and radical scavenging activities (Koksal et al., 2017). Phenolic structures include a diverse group of molecules classified as secondary metabolite in plants which have wide range of structure and functions (Topal et al., 2016). Figure 3 shows the results of TPC evaluation. On the first day of experiments, the lowest amount of TPC was related to the control sample (3.23 mg GAE/g sample). The TPC (2.29 mg GAE/g sample) of fresh ricotta cheese produced by Hamdy and Hafaz (2018) was lower than the TPC obtained for control ricotta cheese in this study. By adding CSE and increasing its level in cheese samples from 1.5% to 3.0%, the TPC increased significantly (*p* < .05). On this day, the TPC of the F‐CSE sample was higher than the E‐CSE cheeses. The F‐CSE 3.0% sample had the highest TPC (38.95 mg GAE/g sample). The presence of bioactive and functional phenolic compounds, including chlorogenic acid, caffeic acid, myricetin, quercetin, and kaempferol, has been reported in chia seeds (Capitani et al., 2012; Rahman et al., 2017). de Falco et al. (2017) and Valdivia‐López and Tecante (2015) also mentioned the presence of caffeic acid, chlorogenic acid, and alpha‐linoleic acid in remarkable amounts in chia seeds as the reason for their noticeable AA. Similar to the result of this study, Kwon et al. (2019) observed that the incorporation of CSE at different levels to the yogurt formulation led to a significant improvement in the TPC of samples compared to control. During the storage period, due to the oxidation of phenolic compounds in cheese samples, the TPC decreased (*p* < .05). Nanoencapsulation of CSE by nanoliposomes and BSG was able to better preserve the phenolic compounds in cheeses compared to the free CSE, which showed consistency with the results obtained by Farrag et al. (2020). These researchers also observed the high stability of encapsulated olive polyphenols extract in cheese samples during cold storage.

### 3.8 AA of cheeses

**FIGURE 4:**
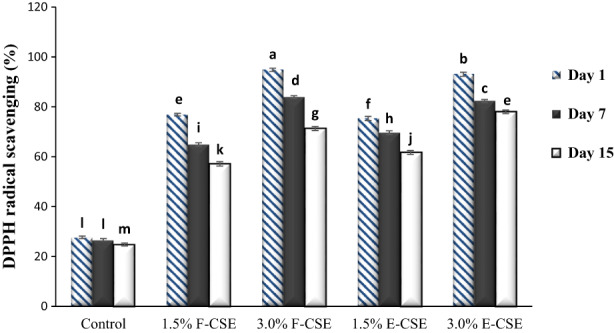
The DPPH radical scavenging of the ricotta cheese samples during 15‐day refrigerated storage period. Bars represent mean (*n* = 3) ± SD. Different letters on the bars indicate significant difference at 5% level of probability among samples. CSE, chia seed extract; DPPH, 2,2‐diphenyl‐1‐picrylhydrazyl; E‐CSE, encapsulated CSE; F‐CSE, free CSE.

**FIGURE 5:**
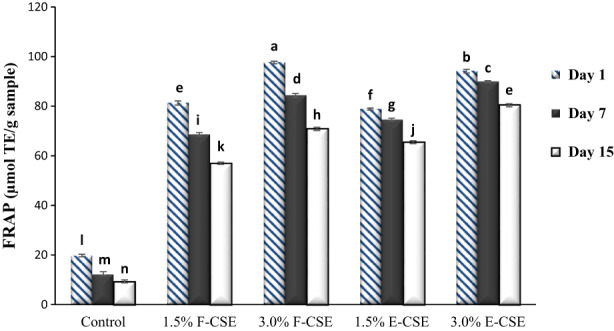
The FRAP of the ricotta cheese samples during 15‐day refrigerated storage period. Bars represent mean (*n* = 3) ± SD. Different letters on the bars indicate significant difference at 5% level of probability among samples. CSE, chia seed extract; E‐CSE, encapsulated CSE; F‐CSE, free CSE; FRAP, ferric reducing antioxidant power.

The AA of ricotta cheeses was measured by two methods and is presented in Figures 4 and 5. On the first day, the control sample had the lowest values of DPPH radical scavenging (27.64%) and FRAP (19.78 μmol TE/g sample). The addition of CSE has increased the AA of samples significantly (*p* < .05). Due to the higher TPC of F‐CSE than E‐CSE, therefore, the F‐CSE samples showed higher AA than E‐CSE samples on the first day (*p* < .05). There was a positive relationship between the concentration of CSE and the AA of cheeses. The cheese sample containing 3.0% CSE had the highest values of DPPH radical scavenging (94.81%) and FRAP (97.62 μmol TE/g sample) on the first day. Over time, the AA of samples decreased (*p* < .05) and the highest rate of decrease was related to the F‐CSE, because as mentioned before, the encapsulation process led to better preservation of phenolic compounds and thus to maintain the AA of cheeses during the storage period. On the last day of experiments, the highest values of DPPH radical scavenging (77.99%) and FRAP (80.36 μmol TE/g sample) were obtained in the cheese samples containing 3.0% encapsulated CSE.

Various natural antioxidants such as carotenoids, tocopherols, and phytosterols have been reported in chia seeds (Ixtaina et al., 2011). Chia seeds are also a rich source of phenolic compounds that play a very important antioxidant role in the AA (Abad & Shahidi, 2020). In the research conducted by Kwon et al. (2019), a significant increase in AA of yogurt samples was observed due to the incorporation of different levels of CSE, and the highest AA (70%) was obtained in the sample containing the highest level of extract. Hamdy and Hafaz (2018) stated that the AA (DPPH) of control ricotta cheese and samples containing thyme, basil, and rosemary were in the range of 26.16%–81.16% on the first day of storage and decreased to 18.23%–63.25% on the last day of storage (21st day). The results of this are also in agreement with those observed by Balabanova et al. (2020) who reported that TPC and AA of Labneh cheese increased significantly due to the addition of encapsulated pepper extracts, and a decrease in the phenol content and AA of cheeses was observed during the storage period.

### 3.9 Peroxide value of cheeses

**FIGURE 6:**
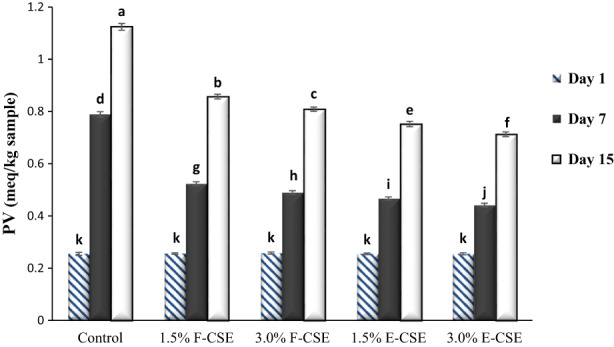
The PV of the ricotta cheese samples during 15‐days refrigerated storage period. Bars represent mean (*n* = 3) ± SD. Different letters on the bars indicate significant difference at 5% level of probability among samples. CSE, chia seed extract; E‐CSE, encapsulated CSE; F‐CSE, Free CSE; PV, peroxide value.

Peroxide value (PV) is an indicator for measuring the primary products of lipid oxidation, including peroxides and hydroperoxides, in food products (El‐Hadary & Taha, 2020). Changes in the PV of ricotta cheese samples containing free and encapsulated CSE are shown in Figure 6. On the first day, the PVs of different cheese samples were in the range of 0.255–0.258 meq/kg sample, and there was no significant difference between the PV of cheeses. During the storage period, due to the development of oxidation reaction of lipids in cheese samples and thus increase the primary oxidative products, the PV of samples significantly increased (*p* < .05). In samples enriched with F‐CSE and E‐CSE, due to the potential AA of this extract, the formation rate of hydroperoxides decreased and the PV showed less increase than the control sample. The increase in CSE causes to increasing in the content of bioactive and functional compounds, especially polyphenols, which led to a decrease in the PV of samples. The PV of ricotta samples on the last day of the storage was in the range of 0.713–1.124 meq/kg sample. Increased PV of various cheeses during the storage period has also been reported by different researchers (Da Silva Dannenberg et al., 2016; Khan et al., 2018; Saravani et al., 2019). Timilsena et al. (2016) also showed that encapsulation of chia seed oil by a complex of chia seed protein and gum increased oxidative stability and decreased the PV of samples during storage period. According to the results of this study, El‐Galeel et al. (2018) showed that over time the PV of soft cheese samples increased significantly; however, the PV was always lower in cheeses enriched with different natural antioxidant extracts (rice bran, potato peel, and peanut skin extracts) than in the control sample. Faion et al. (2015) also found that proto cheese samples containing extracts of mate tea leaves retarded the lipid oxidation process during the repining period.

### 3.10 Sensory evaluation

**FIGURE 7:**
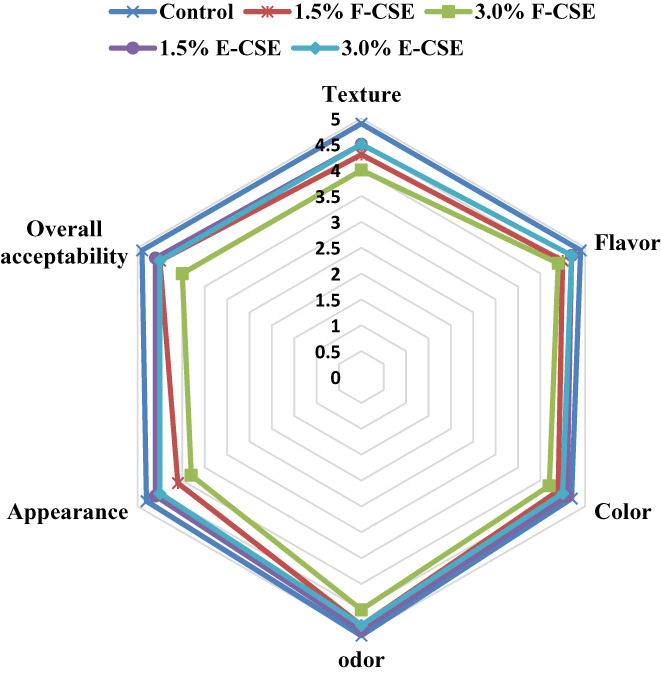
The sensory scores of ricotta cheese samples during 15‐day refrigerated storage period

The results of the sensory evaluation are presented in Figure 7. The addition of the CSE to the cheese formulation caused a decrease in texture, flavor, color, odor, and acceptability scores, but this was significant only in the F‐CSE sample. In terms of appearance, the F‐CSE samples had a significant difference from the control. According to these results, encapsulating CSE was able to reduce the effects of the CSE on sensory characteristics of ricotta cheese. However, despite the decrease in sensory scores due to the addition of free CSE, all samples were acceptable. The treatments did not have an adverse and negative effect on the sensory acceptance. Similar results were observed by El‐Messery et al. (2019) in the incorporation of mandarin peel extract nanoliposomes into processed cheese formula. No adverse effect of adding natural antioxidant extracts including rice bran, potato peel, and peanut skin extracts on sensory characteristics and overall acceptance of soft cheese was also reported by El‐Galeel et al. (2018). Balabanova et al. (2020) indicated higher sensory scores for Labneh cheeses enriched with encapsulated pepper extracts compared to the control cheese.

## 4 CONCLUSION

The results of this study demonstrated that the incorporation of free and encapsulated CSE did not have an adverse effect on the chemical composition, physicochemical properties, and sensory acceptance of ricotta cheese. It was able to remarkability improve the functional activity of cheeses by increasing the TPC and AA. This extract also increased the oxidative stability of cheese samples during the cold storage period. The direct and positive relationship between the TPC and AA of cheeses was confirmed in this research. The nanoencapsulation of CSE also indicated a significant effect on preserving phenolic compounds and improving their stability in cheeses. These results finally showed that 3% encapsulated CSE can be used as a rich source of natural antioxidants to produce functional ricotta cheese with higher oxidative stability and health benefits.

## CONFLICT OF INTEREST

We declare that the results of this study have not been previously published, considered for publication elsewhere, and published by all authors. The authors declare that they have no conflicts of interest to disclose.

## Data Availability

The data that support the findings of this study are available from the author, Farinaz, Hosseini (hosseini_fa@yahoo.com), upon reasonable request.
